# Fungal succession in decomposing ash leaves colonized by the ash dieback pathogen *Hymenoscyphus fraxineus* or its harmless relative *Hymenoscyphus albidus*

**DOI:** 10.3389/fmicb.2023.1154344

**Published:** 2023-04-14

**Authors:** Chatchai Kosawang, Isabella Børja, Maria-Luz Herrero, Nina E. Nagy, Lene R. Nielsen, Halvor Solheim, Volkmar Timmermann, Ari M. Hietala

**Affiliations:** ^1^Department of Geosciences and Natural Resource Management, University of Copenhagen, Frederiksberg, Denmark; ^2^Division of Biotechnology and Plant Health, Norwegian Institute of Bioeconomy Research (NIBIO), Ås, Norway; ^3^Faculty of Environmental Sciences and Natural Resource Management (MINA), Norwegian University of Life Sciences (NMBU), Ås, Norway; ^4^Norwegian Institute of Bioeconomy Research (NIBIO), Steinkjer, Norway

**Keywords:** decomposition, fungal community dynamics, invasive species, *Hymenoscyphus fraxineus*, metabarcoding

## Abstract

**Introduction:**

The ascomycete *Hymenoscyphus fraxineus*, originating from Asia, is currently threatening common ash (*Fraxinus excelsior*) in Europe, massive ascospore production from the saprotrophic phase being a key determinant of its invasiveness.

**Methods:**

To consider whether fungal diversity and succession in decomposing leaf litter are affected by this invader, we used ITS-1 metabarcoding to profile changes in fungal community composition during overwintering. The subjected ash leaf petioles, collected from a diseased forest and a healthy ash stand hosting the harmless ash endophyte *Hymenoscyphus albidus*, were incubated in the forest floor of the diseased stand between October 2017 and June 2018 and harvested at 2–3-month intervals.

**Results:**

Total fungal DNA level showed a 3-fold increase during overwintering as estimated by FungiQuant qPCR. Petioles from the healthy site showed pronounced changes during overwintering; ascomycetes of the class Dothideomycetes were predominant after leaf shed, but the basidiomycete genus *Mycena* (class Agaricomycetes) became predominant by April, whereas *H. albidus* showed low prevalence. Petioles from the diseased site showed little change during overwintering; *H. fraxineus* was predominant, while *Mycena* spp. showed increased read proportion by June.

**Discussion:**

The low species richness and evenness in petioles from the diseased site in comparison to petioles from the healthy site were obviously related to tremendous infection pressure of *H. fraxineus* in diseased forests. Changes in leaf litter quality, owing to accumulation of host defense phenolics in the pathogen challenged leaves, and strong saprophytic competence of *H. fraxineus* are other factors that probably influence fungal succession. For additional comparison, we examined fungal community structure in petioles collected in the healthy stand in August 2013 and showing *H. albidus* ascomata. This species was similarly predominant in these petioles as *H. fraxineus* was in petioles from the diseased site, suggesting that both fungi have similar suppressive effects on fungal richness in petiole/rachis segments they have secured for completion of their life cycle. However, the ability of *H. fraxineus* to secure the entire leaf nerve system in diseased forests, in opposite to *H. albidus*, impacts the general diversity and successional trajectory of fungi in decomposing ash petioles.

## Introduction

Common ash (*Fraxinus excelsior* L) is threatened across Europe by the invasive ascomycete *Hymenoscyphus fraxineus* (syn. *H. pseudoalbidus*; anamorph *Chalara fraxinea*; Kowalski, 2006; Queloz et al., 2011; Baral et al., 2014). This fungus is considered to originate from Asia, where it is a leaf endophyte associated with local ash species that are phylogenetically closely related to common ash (Inoue et al., 2019). In common ash, the fungus causes shoot dieback that results in gradual crown decline and eventual tree death. The first observation of ash dieback is from 1992 in Poland, and during the following decades, the disease spread practically throughout the natural distribution range of common ash (Solheim and Hietala, 2017). The current disease cycle model suggests that the pathogen’s wind-borne ascospores produced by fruiting bodies, formed on shed ash leaf tissues after overwintering, germinate on the leaf surface to give rise to leaf infection, which in turn is followed by mycelial spread through the petiole into shoots and twigs prior to leaf shed (Haňáčková et al., 2017). Besides the leaf-to-shoot route, lenticels (Nemesio-Gorriz et al., 2019) and direct penetration of epidermis of young shoots (Mansfield et al., 2019) may offer additional entrances for pathogen ascospore germlings to the stem tissues. The disease is manifested by local necroses of leaf tissues, wilting of leaves and young shoots, premature leaf shed, bark necrosis and crown dieback (e.g., Przybył, 2002).

As many as 10,000 ascomata of *H. fraxineus* can be present per m^2^ forest floor in stands with epidemic level of ash dieback (Hietala et al., 2013). This causes a tremendous propagule pressure, something that is typical of invasive species, and which has obviously facilitated the rapid spread of the disease across Europe. An annual spread rate of up to 75 km has been estimated for ash dieback in Europe (Gross et al., 2014; Solheim and Hietala, 2017). Mortality rates of up to 80% have been recorded in common ash forests (Coker et al., 2019). Many species are obligately or with high affinity associated with common ash, and the disappearance of ash poses a set of cascading impacts upon the biodiversity associated with this keystone tree species (Mitchell et al., 2014).

The “Diversity Resistance” hypothesis argues that diverse natural communities are highly competitive and readily resist invasion based on the assumption that such communities are structured by interspecific competition in which niche space is a limiting factor (Levine and D’Antonio, 1999; Laforest-Lapointe et al., 2017). The leaf niche is spatially, temporally, and nutritionally heterogeneous. Nutrition for microbes is available on leaf surface in the form of exudates leaking from the tissues and internally in the form of structural carbohydrates, energy-storage compounds, and secondary metabolites such as host defense compounds (Fonseca and Inacio, 2006). In deciduous trees, the amount and composition of the latter two nutrient pools can be envisaged to vary during the season and depend also on the tree health status and canopy position. The microbial species richness generally increases with leaf age (Sieber, 2007). Chance events, such as composition of the microbial propagule pool at the habitat and deposition of spores in a favorable substrate and microenvironment for germination, shape microbial communities. However, the assembly of coexisting species is also determined by interspecific trade-offs in the abilities of species to deal with the factors that constrain their fitness and abundance (Tilman, 2000). Like other angiosperm trees, leaves of common ash host a wide range of phylogenetically diverse fungi that include both polyphagous species and species with high affinity to ash (Cross et al., 2017; Schlegel et al., 2018; Becker et al., 2020).

In Norway, the peak ascospore production of *H. fraxineus* occurs commonly during the second half of July and first half of August (Timmermann et al., 2011; Hietala et al., 2013). By that time, the leaf tissues of common ash have already been colonized by a wide range of fungi that, depending on their mode of feeding, reside either on leaf surface (epiphytes), as small dormant endophytic thalli in leaf epidermis/petiole cortex (endophytes) or as intracellular haustoria in leaf blade mesophyll (biotrophs; Cross et al., 2017). *Hymenoscyphus fraxineus* colonizes primarily the starch-rich parenchyma and phloem cells in the vascular cylinder of leaf veins (Nielsen et al., 2022), cell types that are normally free of infection. The parenchyma cells respond to *H. fraxineus* colonization by accumulation of phenolic compounds. Although the accumulation does not seem to prevent the growth of this fungus (Nielsen et al., 2022), it obviously causes a qualitative change in the carbon sources present in leaves.

Fungal community dynamics in decomposing ash leaf litter in healthy stands or stands infested by *H. fraxineus* have received little attention so far. Genome analysis indicated that both *H. fraxineus* and *Hymenoscyphus albidus*, a harmless indigenous associate in leaves of common ash, harbor an extensive repertoire of cell wall carbohydrate active enzymes and appear better equipped for saprobic feeding than *Botrytis cinerea* and *Sclerotinia sclerotiorum*—necrotrophic members of Helotiales (Stenlid et al., 2017). *Hymenoscyphus fraxineus* can survive as pseudosclerotia in infected leaf petioles for several years before fruiting body formation (Kirisits, 2015), testifying for a strong ability to defend the saprotrophic niche. It is noteworthy that *H. fraxineus* and *H. albidus* differ in the colonization degree of ash leaf vein tissues. The pseudosclerotial plates involved in securing the saprotrophic niche are relatively small for *H. albidus* in comparison to those of *H. fraxineus*, which typically extend throughout the entire petiole and rachis system (Baral and Bemmann, 2014) and even throughout the nerves of leaflets (see Figure 2 in Hietala et al., 2013).

The stochastic model of community assembly predicts that successful invaders should decrease the abundances of species that are competitively close to them on the trade-off surface (Tilman, 2004). We hypothesized that the successional changes in fungal community during decomposition of ash leaves differ between healthy forests and forests with epidemic level of ash dieback. To pursue this, we used high throughput sequencing of internal transcribed spacer 1 (ITS1) region in the eukaryotic ribosomal cluster to profile fungal community in petiole/rachis tissues, naturally infected by either *H. fraxineus* or *H. albidus*, during the first 8 months after leaf shed.

## Materials and methods

### Plant material

Shed leaves of common ash were collected at two sites in Norway during the last week of October in 2017. The first site, a natural forest located on a steep north-west facing slope in central Norway in Stjørdal municipality (63°26′46.84806″N, 10°59′7.71109″E, 5–30 m a.s.l), showed no signs of ash dieback at the time of sampling. Ash is the canopy forming tree species at the stand with the largest trees up to 20 m in height. In addition, ash is present in the understory along with some saplings of rowan (*Sorbus aucuparia* L.), aspen (*Populus tremula* L.), bird cherry (*Prunus padus* L.), alder (*Alnus* spp.) and willow (*Salix* spp.). This healthy site is referred to as the Stjørdal site in the text. The second site, located in south-east Norway (Ås municipality, 59°40′44″N, 10°46′31″E, 100 m a.s.l), has a several-year-long disease history of ash dieback and has been subject of several prior studies on ash dieback (Timmermann et al., 2011; Hietala et al., 2013; Agan et al., 2020). The site is a naturally regenerated moist forest with rich understory vegetation dominated by meadowsweet (*Filipendula ulmaria* (L.) Maxim.). Ash is present both as a canopy forming tree (the largest ash trees ranging between 20 and 27 m in height) and in the understory along with rowan, aspen, bird cherry, downy birch (*Betula pubescens* Ehrh.), alder and willows. This diseased site is referred to as the Norderås site in the text.

The leaflets and leaflet petioles were removed upon collection of shed leaves. In October, the petiole/rachis tissues from Stjørdal were light-colored with some occasional small areas with brown discoloration. Approximately half of the petioles from Norderås had a similar appearance as those from Stjørdal, while the rest had relatively large areas of discoloration (Supplementary Figures 1A,B). The petiole/rachis tissues, referred to as petioles later in the text, were placed in labeled nylon litterbags (1 mm nylon mesh size)—24 bags with 15 petioles in each bag. Twelve of the bags contained petioles from the Stjørdal site and the other 12 contained petioles from the Norderås site. The weight of plant material in each nylon bag was recorded after an incubation of 2 days at room temperature. For control purpose, three nylon bags with plant material from each site were placed in −20°C freezer on 31st October 2017. These bags were referred to as October-collected samples in further text. The same day, the remaining nine nylon bags with petioles from each site were placed on the ground in a 2 m × 2 m plot at the Norderås site. The bags were anchored to the ground with labeled sticks and covered with leaf litter (Supplementary Figures 1C,D). Three bags with petioles from each site were collected on 30th January, 16th April, and 18th June 2018 and stored at −20°C until further processing. Together with the samples collected in October 2017, these samples represented four different seasons—autumn (October), winter (January), spring (April), and summer (June).

For comparison, five petioles with *H. albidus* ascomata, collected from the healthy site in August 2013 and kept at −20°C, were divided in two types of segments. Type 1 segments contained a fully developed pseudosclerotium around the petiole circumference (referred to as dark area later in the text), while type 2 segments either showed no pseudosclerotium or only part of the petiole circumference was covered by one pseudosclerotium (referred to as light area later in the text; Supplementary Figure 2). Before DNA extraction, *H. albidus* ascomata were removed, and the two types of segments were processed separately.

### Extraction of DNA from the plant material and qPCR

The petioles from each nylon bag were dried at 28°C for 48 h after which the dry weight was recorded. Three randomly selected petioles per nylon bag were pooled together, frozen with liquid nitrogen, and pulverized with Retsch 300 mill (Retsch GmbH, Germany) using liquid nitrogen-cooled stainless-steel containers and beads. For DNA isolation, 20–25 mg of tissue was processed with DNeasy Plant Mini Kit (Qiagen, Germany) according to the manufacturer’s instructions. The reference petiole segments with or without *H. albidus*’s pseudosclerotia were processed identically unless otherwise stated. The obtained DNA was quantified using NanoDrop 2000 (Thermo Scientific, United States).

The forward primer FungiQuant-F (5′-GGRAAACTCACCA GGTCCAG-3′), the reverse primer FungiQuant-R (5′-GSWCTAT CCCCAKCACGA-3′) and the probe FungiQuant-Prb (5′-FAM-TGG TGCATGGCCGTT-BHQ1-3′; Liu et al., 2012) were used for quantification of total fungal DNA. For detection of *H. fraxineus*, the forward primer Cfrax-F (5′-ATTATATTGTTGCTTTAGCAGGTC-3), the reverse primer Cfrax-R (5′-TCCTCTAGCAGGCACAGTC-3′) and the probe Cfrax-P (5′-FAM-CTCTGGGCGTCGGCCTCG- BHQ1-3′; Ioos et al., 2009) were used. Primer and probe concentrations were 300 and 100 nM, respectively, for both assays. For detection of *H. albidus*, we used the primer probe set designed and tested for species specificity by Husson et al. (2011) with the modification of using JOE as the reporter dye (Hietala et al., 2013). The forward primer Halb-F (5′TATATTGTTGCTTTAGCAG GTCGC-3′), the reverse primer Halb-R (5′-ATCCTCTAGCAGGCA CGGTC-3′) and the probe Halb-P (5′-JOE-CCGGGGCGTTGGC CTCG-BHQ1-3′) were all used at the concentrations of 900 nM (Hietala et al., 2013). All amplifications were performed in Takyon™ Low Rox Probe MasterMix dTTP Blue (Eurogentech, Belgium) according to manufacturer instructions on an Applied Biosystems ViiA 7 qPCR machine (ThermoFisher Scientific, United States). PCR cycling parameters were 95°C for 10 min, followed by 40 cycles of 95°C for 15 s and 65°C for 55 s for all the above-mentioned assays. Three-log dilution series were prepared for all the experimental samples. Each experimental sample had undiluted DNA as the most concentrated and all log dilutions of a sample were used as templates in real-time PCR. Three microliters of the DNA solution were used as the template for each 25-μl reaction. For all real-time PCR assays, each time point had three biological replicates and each biological replicate was repeated twice. Standard curves for DNA quantity for the *Hymenoscyphus* species were constructed based on DNA extracted from pure cultures of *H. fraxineus* and *H. albidus*. We used an absolute quantification approach to calculate the DNA amount of these two fungi in the experimental samples.

### PCR and ITS1 amplicon sequencing

The primer pair BITS (5′-ACCTGCGGARGGATCA-3) and B58S3 (5′-GAGATCCRTTGYTRAAAGTT-3′; Bokulich and Mills, 2013) containing appropriate Illumina Nextera adapters and barcodes was used to amplify the Internal Transcribed Spacer 1 (ITS1) region of fungal species associated with the petioles. Each 25-μL PCR mixture contained 25 ng of DNA, one unit of AccuPrime Taq DNA Polymerase, high fidelity (Invitrogen, United States) and 0.2 μM of each primer. PCR was set up on a Bio-Rad T100 thermal cycler (Bio-Rad, United States) as follows: 94°C for 2 min followed by 32 cycles of 94°C for 30 s, 54°C for 30 s, and 68°C for 30 s, with a final extension at 68°C for 3 min. PCR for each sample were done in triplicate and subjected to PCR clean-up, pooling and sequencing with Illumina MiSeq V3 chemistry (2 × 300 bp) at the Integrated Microbiome Resource, Dalhousie University, Canada.

### Data processing and statistical analysis

For petiole weight loss, total DNA amount and FungiQuant Cq values, one-way ANOVA coupled with Tukey *post hoc* test was used to consider the significance of differences between petioles from the two sites. Owing to the low number of replicates, the data for the different time-points was pooled. Linear regression and the coefficient of determination (r^2^) were calculated to consider the relationships between weight loss, total DNA amount and FungiQuant Cq values. For Illumina data, low-quality bases (Q < 20), short reads (<75 bases) and primer sequences were discarded using BBDuk in the BBTools package version 38.90 and q2-cutadapt plugin of Cutadapt (Martin, 2011), respectively. VSEARCH (Rognes et al., 2016) was used for paired-end read merging, chimera filtering and closed-reference Operational Taxonomic Unit (OTU) picking at 99% similarity threshold against the UNITE ITS database version 8 (Nilsson et al., 2019). Spurious OTUs with low frequencies (<0.005% of total sequences) were excluded from further analyses following Bokulich et al. (2013).

All samples were rarified to 8,557 reads per sample. Alpha (Chao1, Observed OTUs, Shannon and Simpson) and beta (Bray–Curtis dissimilarity) diversity indices were calculated for each sample using the rarefied datasets. Kruskal-Wallis tests and permutational multivariate analysis of variance (PERMANOVA) with 9,999 permutations were applied to test for significant differences in alpha- and beta-diversity estimates within and between samples from Stjørdal and Norderås for each collection time. The same tests and analyses were applied to compare communities associated with the dark pseudosclerotial and neighboring light areas of the reference petioles with *H. albidus* ascomata, collected in Stjørdal in August 2013. LEfSe (linear discriminant analysis effect size; Segata et al., 2011), implemented through microbiomeMarker (Cao et al., 2022), was used to compare taxonomic profiles of the fungal communities associated with Stjørdal and Norderås petioles. LEfSE was also applied to detect differentially abundant taxa (OTUs) between time points within each site, and for each time point between the two sites (CPM-normalized count data with a LDA cutoff of 4). FDR correction (Benjamini and Hochberg, 1995) was applied and taxa were called significantly abundant at FDR correction ≤ 0.05. FUNGuild (Nguyen et al., 2016) was used to predict ecological guild for each OTU. The predicted guilds were accepted only at highly probable or probable levels as recommended by the authors.

All the bioinformatic and statistical work, except the removal of low-quality bases and principal coordinate analysis (PCoA), was carried out in QIIME2 space (version 2021.11; Bolyen et al., 2019). The R package PhyloSeq (McMurdie and Holmes, 2013) and R version 4.2.1 (R Core Team, 2018) were used for PCoA analysis, while data visualization was carried out using either PhyloSeq in R or Graphpad Prism version 9 (Graphpad Software, United States).

## Results

### Petiole weight loss and changes in fungal DNA level during overwintering

**Figure 1:**
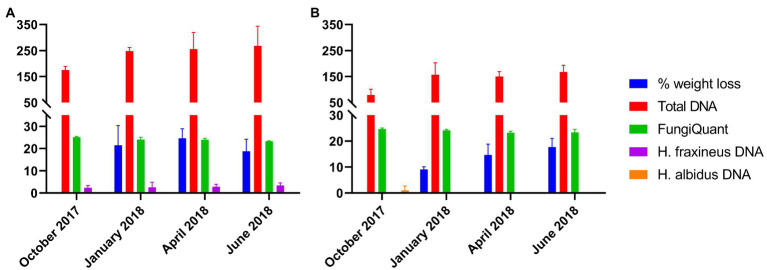
Petiole weight loss (%), total DNA amount (measured by Nanodrop, ng of DNA/mg of tissue), estimate of fungal DNA level (measured by FungiQuant qPCR, Cq value) and DNA amounts (ng of DNA/mg of tissue) of *Hymenoscyphus fraxineus* and *Hymenoscyphus albidus* as estimated by species-specific qPCR assays over the four time points. The data was collected from samples from Norderås **(A)** and Stjørdal **(B)**. Bars indicate standard deviation, *n* = 3 per time point.

Weight loss increased continuously over the experimental period for petioles from Stjørdal, while for petioles from Norderås maximum weight loss was recorded in April (Figure 1). The average weight loss recorded for petioles from Norderås during overwintering was significantly higher (*p* < 0.0001) than that recorded for petioles from Stjørdal (21.6% and 13.8%, respectively). Petioles from both sites showed a continuous increase in total DNA amount (as estimated by Nanodrop; Supplementary Data 1) during the experimental period. The average DNA amount in petioles from Norderås was significantly higher (*p* = 0.0001) than in petioles from Stjørdal (237 and 139 ng/mg tissue, respectively). The coefficient of determination (r^2^) between weight loss and total DNA amount was 0.51 for petioles from Stjørdal (*p* = 0.009) and 0.47 (*p* = 0.014) for petioles from Norderås. In both sites, FungiQuant qPCR indicated a continuous increase in fungal DNA amount during the experimental period. The mean Cq value declined from 25.1 to 23.3 and from 24.7 to 23.3 in the material from Norderås and Stjørdal, respectively. The difference in the Cq values between the two materials was not significant. The correlation between FungiQuant Cq values and weight loss was higher for petioles from Stjørdal than for petioles from Norderås, with respective r^2^ values of 0.66 (*p* = 0.001) and 0.24 (*p* = 0.1). The relation between FungiQuant Cq values and total DNA amount was also clearer for petioles from Stjørdal than for petioles from Norderås, with respective r^2^ values of 0.40 (*p* = 0.028) and 0.09 (*p* = 0.34). The amount of *H. fraxineus* DNA, as determined by a species-specific qPCR, showed a slight and non-significant increase in petioles from Norderås during the experimental period. The r^2^ values between *H. fraxineus* DNA amount and weight loss, FungiQuant Cq values and total DNA amount were 0.01 (*p* = 0.74), 0.02 (*p* = 0.64) and 0.19 (*p* = 0.56), respectively, for petioles from Norderås. A low level of *H. albidus* DNA was detected in one replicate of material from Stjørdal (Figure 1).

### Fungal species composition and changes during overwintering

The amplicon sequencing of ITS1 produced approximately 1.2 million high-quality reads for 24 samples, giving between 8,557 and 141,468 reads per sample. For all samples, the alpha rarefaction curves of Chao1, observed OTUs, Shannon and Simpson indices appeared to approach a plateau, suggesting that only rare species may remain undetected (Supplementary Figure 3). We identified a total of 168 OTUs present in the petioles from both sites; 96 (57.14%) and 51 (30.35%) of these belonged to Ascomycota and Basidiomycota, respectively. A list of the detected OTUs with their corresponding species hypothesis codes is shown in Supplementary Data 2. Twenty-one (12.5%) OTUs were assigned to unidentified fungi. No fungal taxa in any other phyla were noticed in the observed communities. Of the 168 OTUs, FUNGuild was able to identify guilds of 67 (39.8%) OTUs at either probable or highly probable level of confidence. The majority of identified OTUs were predicted to be saprotrophs (26 OTUs, 38.8%) such as *Crocicreas cyathoideum*, *Gyoerffyella craginiformis* and *Tetracladium furcatum*, followed by pathotroph-saprotrophs (19 OTUs, 28.3%) such as *Didymella macrostoma* and *Leptospora rubella*, and pathotrophs (17 OTUs, 25.3%) such as *Mycosphaerella tassiana* and *Phyllactinia fraxini*. Only three, one and one OTUs were predicted to be pathotrophs-saprotrophs-symbiotrophs, a saprotroph-symbiotroph, and a symbiotroph, respectively (Supplementary Figure 4; Supplementary Data 3).

**Figure 2:**
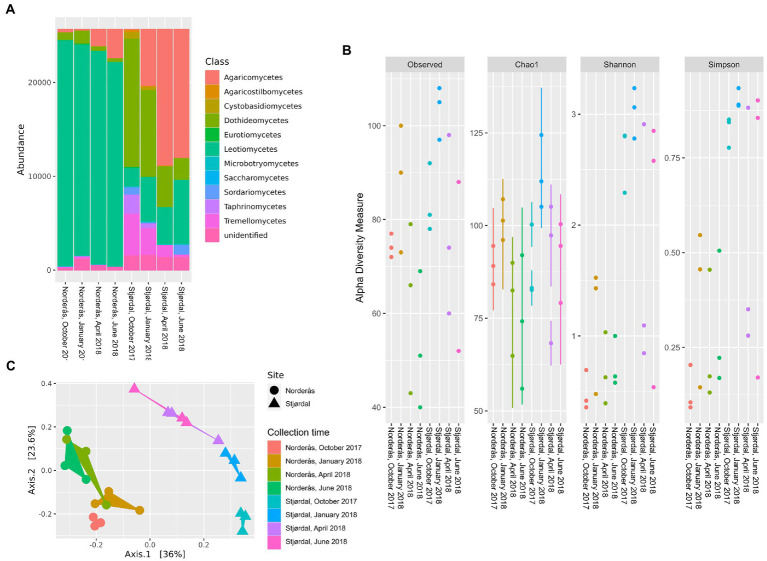
**(A)** Class-level distribution of the petiole-associated fungal communities from the diseased (Norderås) and healthy (Stjørdal) sites over four collection time points. **(B)** Alpha diversity indices of petiole mycobiomes from the diseased (Norderås) and healthy (Stjørdal) sites, and **(C)** principal coordinate analysis (PCoA) based on Bray–Curtis dissimilarity matrices showing the separation of mycobiomes from each site and season.

**Figure 3:**
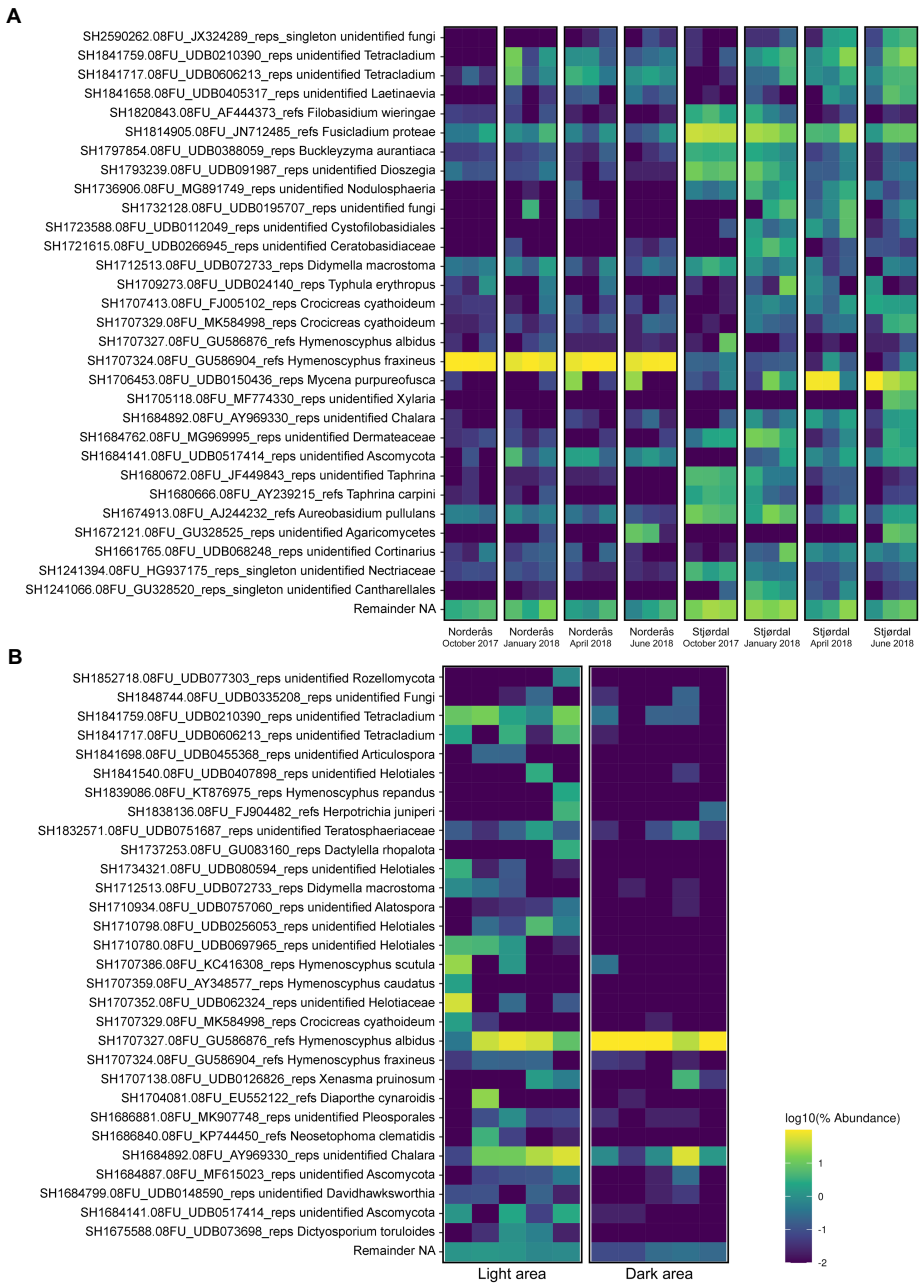
Top 30 highly abundant OTUs associated with **(A)** petioles from the diseased (Norderås) and healthy (Stjørdal) sites during overwintering in the period 2017–2018 and **(B)** the dark pseudosclerotial and adjacent light petiole areas from Stjørdal samples that hosted *Hymenoscyphus albidus* ascomata upon collection in 2013.

Concerning the phylum Ascomycota, its read proportion decreased from 97.5% to 87.4% and from 73.1% to 42.1% between October and June in the petioles from Norderås and Stjørdal, respectively (Figure 2A). While the read proportion of class Leotiomycetes declined from 93.7% in October to 84.9% in June in the petioles from Norderås, the read proportion of the class increased from 7.6% to 26.6% in the Stjørdal petioles. *Hymenoscyphus fraxineus* (order Helotiales), which constituted most of the reads of Leotiomycetes in the Norderås petioles, had read proportions of 93, 77.1%, 85.1%, and 81.8% in October, January, April, and June, respectively. In the Stjørdal petioles, its read proportions remained low, between 0.1% and 0.5%, during overwintering (Figure 3A). A low read proportion of *H. albidus* was observed both in the Norderås (between 0% and 0.1%) and Stjørdal material (0%–3.0%) during overwintering (Figure 3A). In June, other members of the Helotiales order, namely genera *Tetracladium*, *Crocicreas*, *Laetinaevia*, and *Chalara*, constituted 2.0% of the reads in the Norderås petioles and 21.3% of the reads in the Stjørdal petioles. Fungi in the class Dothideomycetes were the predominant Ascomycota in the Stjørdal petioles in October, but their read proportion declined steadily during overwintering, from 53.3% to 9.0%. In the Norderås petioles, the read proportion of the class declined from 3.1% to 1.5% over the winter. In material from both sites, *Fusicladium proteae* (order Venturiales) and *Aureobasidium pullulans* (order Dothideales) were the most common species of Dothideomycetes in October. The read proportions of *F. proteae* between October and June declined from 1.0% to 0.5% and from 38.4% to 6.1% in the petioles from Norderås and Stjørdal, respectively. The read proportions of *A. pullulans* declined between October and June from 0.4% to 0.1% and from 7.9% to 1.2% in the petioles from Norderås and Stjørdal, respectively (Figure 3A). Fungi in the class Taphrinomycetes, order Taphrinales, were the second most common ascomycetes in the Stjørdal petioles in October, with a read proportion of 8%, but by June their read proportion had declined to 0.1%, a level comparable to that observed in the petioles from Norderås during the entire monitoring period. Ascomycetes in the class Sordariomycetes, order Xylariales, were recorded at a relatively high proportion (4.2%) in June in the Stjørdal petioles, whereas they were absent in the Norderås petioles.

Concerning the phylum Basidiomycota, its read proportion in the period between October and June increased from 2.1% to 12.4% in the Norderås petioles and from 21.8% to 54.6% in the Stjørdal petioles (Figure 2A). Fungi in the class Agaricomycetes were the predominant basidiomycetes by June, their read proportion increasing from 1.4% to 12.2% in the Norderås petioles and from 0.4% to 53.6% in the Stjørdal petioles. *Mycena purpureofusca* in the order Agaricales was the predominant fungus in this class by June in both plant materials, with read proportion increasing between October and June from <0.1% to 7.4% and from 0.2% to 47.6% in the petioles from Norderås and Stjørdal, respectively (Figure 3A). In contrast, the read proportions of basidiomycetous yeasts (orders Agaricostilbales, Cystofilobasidiales, Filobasidiales, Sporidiobolales, and Tremellales) declined between October and June, from 0.6% to 0.1% and from 18% to 1% in the petioles from Norderås and Stjørdal, respectively.

The levels of community richness and evenness were distinct between the two sites. For both sites the indices (observed OTUs, Chao1, Simpson and Shannon) were higher in January than in the other time points, and generally higher in the Stjørdal communities than in the Norderås communities (Figure 2B). The Kruskal–Wallis tests showed significant differences in the number of observed OTUs (*p* = 0.03), Simpson index (*p* < 0.01) and Shannon index (*p* < 0.01) between the communities from Norderås and Stjørdal, but not for Chao1 (*p* = 0.14). The subsequent pairwise comparison of Shannon and Simpson diversity indices from the Norderås and Stjørdal communities revealed significant differences for October and January communities (*p* = 0.049 in both cases and for both indices). Principal Coordinates Analysis on Bray-Curtis dissimilarities identified explicit structures. The fungal communities in petioles tended to cluster together according to site and season, but the magnitude of seasonal changes was potentially more pronounced in the Stjørdal material (Figure 2C). PERMANOVA analysis confirmed significant differences in fungal communities in petioles from the two locations (*p* < 0.01). Although we observed significant effects of season (time of collection) for fungal communities in the Stjørdal petioles (*p* < 0.01), the effects were insignificant in the Norderås petioles (*p* = 0.37).

LefSe analysis of the Stjørdal and Norderås communities for the same time point showed that the Stjørdal petioles were significantly enriched with various Ascomycetes and Basidiomycetes, while the Norderås petioles were significantly enriched with only Ascomycetes. Abundant taxa in the Stjørdal petioles comprised the genera *Aureobasidium*, *Buckleyzyma*, *Fusicladium* and *Filobasidium* in October and January, the genera *Alternaria*, *Fusicladium* and *Cystofilobasidiales* in April and the genera *Fusicladium* and *Crocicreas* in June. The petioles from Norderås were significantly enriched with *H. fraxineus*, among others. We also compared the taxonomic profiles across all seasons within each site. While there were no differently abundant taxa in the diseased site, the family Ceratobasidiaceae and the genus *Crocicreas* were over-represented in the petioles from Stjørdal in January and the genus *Laetinaevia* in June, respectively. We did not find any fungal taxa enriched in the petioles from Stjørdal in April. A complete list of the differential abundant taxa identified by LFfSe is provided in Supplementary Data 4.

### Fungal species composition in petioles with *Hymenoscyphus albidus* ascomata

**Figure 4:**
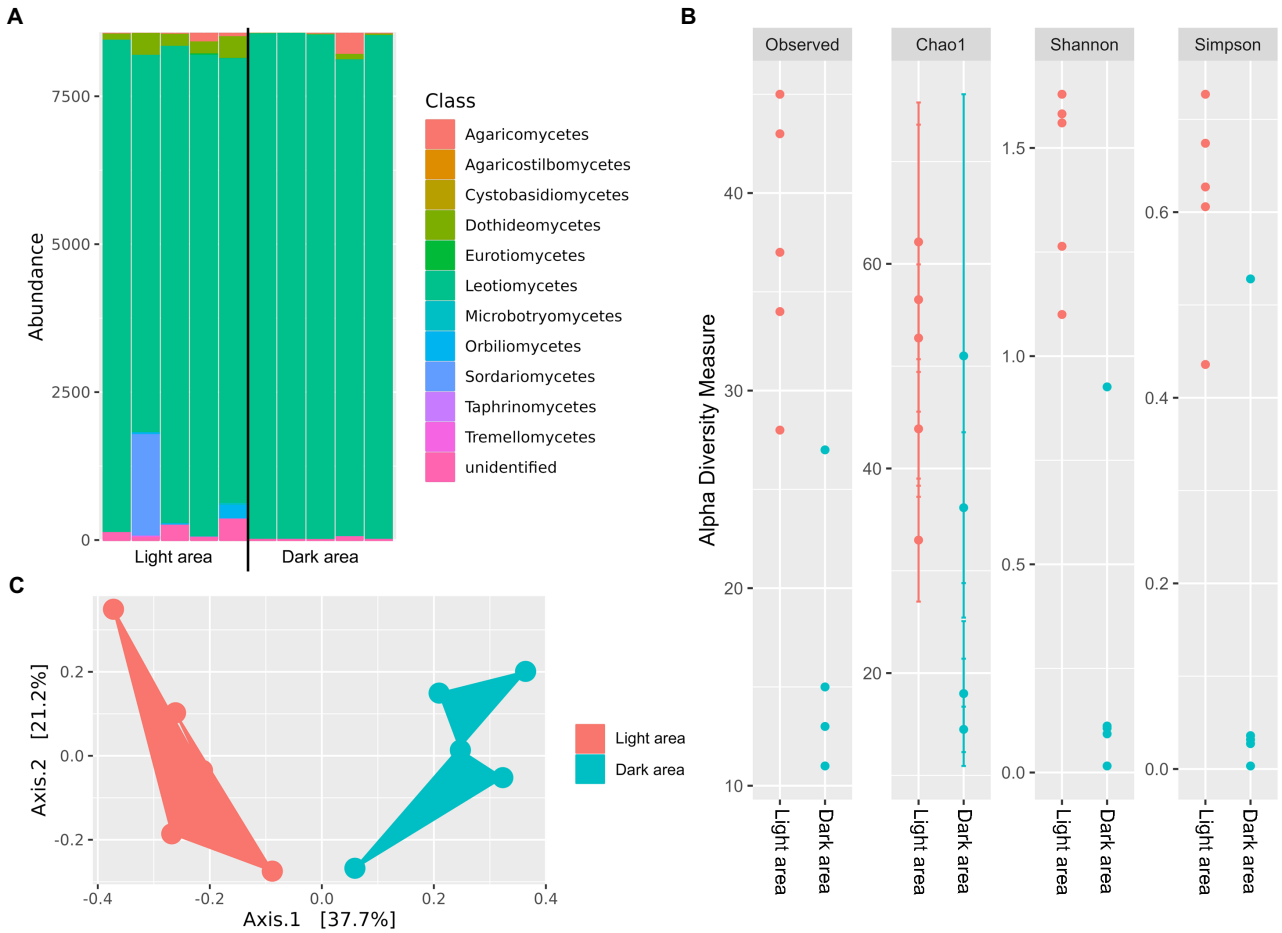
**(A)** Class-level distribution of the mycobiomes associated with the dark pseudosclerotial and the adjacent light reads of the petioles collected from Stjørdal in 2013, **(B)** alpha diversity indices of the mycobiomes from the two areas and **(C)** PCoA analysis based on Bray–Curtis dissimilarity matrices of the dark and light petiole area communities.

We further profiled fungal species composition in the dark pseudosclerotial and adjacent light areas in petioles with naturally formed *H. albidus* ascomata sampled in Stjørdal, 2013 (Figure 3B). A total of 446,033 reads (min. 15,364 reads/sample, max. 116,779 reads/sample) were generated for 10 samples (five samples from each area) and 95 OTUs were retrieved from these samples. Of these, 62 (65.3%) and 23 (24.2%) OTUs belonged to Ascomycota and Basidiomycota, respectively. One (1.0%) OTU represented Rozellomycota, a clade with an unresolved taxonomic position, while the remaining nine (9.5%) OTUs were unidentified. Within Ascomycota, fungi in the class Leotiomycetes, Sordariomycetes, and Dothideomycetes were the three most prevalent taxa with read proportions corresponding to 94.2%, 2.0%, and 1.6%, respectively. The other Ascomycota included Eurotiomycetes, Orbiliomycetes, and Taphrinomycetes, but these taxa together comprise roughly 0.3% of total reads. Basidiomycota also shared only a small fraction with only 0.8% of read proportion. Agaricomycetes and Tremellomycetes were the two most frequently observed Basidiomycota (Figure 4A). FUNGuild assigned similar trophic profiles of the specialized communities associated with the dark pseudosclerotial and the light adjacent areas as to those of the whole petiole communities. Of the 45 OTUs identified at highly probable or probable level, species with saprotrophic lifestyle (21 OTUs) was the largest group followed by those with pathotrophic-saprotrophic (12 OTUs) and pathotrophic (6 OTUs) ones (Supplementary Data 3).

In regions with pseudosclerotia, *H. albidus* (SH1707327.08FU_GU586876_refs) was the predominant species with a read proportion up to 86%. Further analysis using LEfSe confirmed that the species was significantly enriched in the pseudosclerotial area compared to the adjacent light area (*p* < 0.05; Supplementary Data 4). Other prevalent species included an unidentified *Chalara* sp. and the basidiomycete *Xenasma pruinosum* (order *Polyporales*), with read proportions of 12.4% and 0.8%, respectively (Figure 3B). In the light areas, *H. albidus* had a read proportion of 36%, followed by species of *Chalara*, unidentified *Helotiaceae* and *Tetracladium*, with read proportions of 22.7%, 10.3%, and 9.8%, respectively. *Xenasma pruinosum* was the most common basidiomycete with a read proportion of 0.4%. A small fraction of *H. fraxineus* was recorded from both dark and light samples (0.01% of read proportion from the dark area and 0.1% of read proportion from the light area).

The alpha diversity indices (Observed OTUs, Chao1, Simpson and Shannon) were significantly higher (*p* < 0.01 for Observed OTUs, Simpson and Shannon, and *p* < 0.05 for Chao1) in the light area than the dark pseudosclerotial area (Figure 4B). PCoA on Bray-Curtis dissimilarities also depicted two separated clusters of the communities (Figure 4C), suggesting that the two communities are of distinct structures. PERMANOVA analysis confirmed that the structure of communities associated with the pseudosclerotial and adjacent light areas differed significantly (*p* = 0.04). We also applied PERMANOVA to compare the community associated with the two specialized areas with those associated with the entire petiole samples from both Norderås and Stjørdal. Significant differences between the community structures of either dark or light area and those of petioles from Norderås or Stjørdal were observed irrespective of season (*p* ≤ 0.02 for all comparisons).

## Discussion

The alien ash dieback pathogen *H. fraxineus* is obviously highly dependent on saprotrophic competence for its invasiveness in Europe, as it spreads *via* airborne ascospores produced by ascomata formed in decomposed ash leaf petioles. Besides physicochemical properties of the organic material subjected to decomposition and traits of the associated microbial species, temperature is a main determinant of microbial activity (e.g., Davidson and Janssens, 2006; Hietala et al., 2015). To account for potential environmental factors that could impact fungal community dynamics, we subjected common ash petioles collected from an ash forest infested by *H. fraxineus* and a healthy ash forest hosting *H. albidus*, a harmless fungus indigenous to Europe, to overwintering in the diseased site as part of our investigation into the successional changes that take place in fungal community structure during petiole decomposition. Although both petiole materials showed a similar continuous increase in total fungal DNA amount across the overwintering period, as measured by FungiQuant (Liu et al., 2012), the differential abundance and dynamics of ascomycetes and basidiomycetes between petioles from the healthy and diseased stands present challenges for comparing fungal biomass levels. Basidiomycetes tend to have larger genomes and higher copy numbers of rRNA gene cluster than ascomycetes (Lofgren et al., 2018). Additionally, the number of nuclei per cell can vary considerably between different species, from one to several dozen (e.g., James et al., 2008).

The observed dominance of ascomycetes in the class Dothideomycetes in petioles from the healthy site at the time of leaf shed is consistent with the findings of a German study based on fungal isolation from ash leaf tissues in a healthy forest (Reiher, 2011). The now recorded rapid decline in the sequence read proportion of Dothideomycetes in petioles from the healthy site during overwintering and the concomitant increases of the ascomycete class Leotiomycetes and the basidiomycete class Agaricomycetes would fit well with the general model that after leaf senescence ascomycetous leaf endophytes switch to saprophytic feeding to metabolize labile biopolymers, but become gradually replaced by basidiomycetes that decompose more recalcitrant compounds (Promputtha et al., 2007; Voříšková and Baldrian, 2013; Vivelo and Bhatnagar, 2019). The fungal community composition and changes recorded during overwintering in petioles from the healthy site would seem to follow the trajectory model presented by Frankland (1998), which described that Dothideomycetes are dominant in senescent ash leaves, while basidiomycetes colonize ash leaf debris within 6 months of leaf shed.

Concerning forests infested by *H. fraxineus*, we are not aware of any prior study that would have monitored changes in fungal community along decomposition of ash leaf debris. The observed predominance of Leotiomycetes (primarily *H. fraxineus*) across overwintering and increase in read proportion of Agaricomycetes toward summer would, at fungal phylum level, seem to be rather comparable to the finding report by Kowalski and Bilański (2021). These authors isolated fungi from surface-sterilized petioles collected from diseased ash forests in Poland in May and June the year following leaf shed. The study-specific differences at fungal species level can relate to multiple factors. Habitats with different local edaphic conditions and plant communities can also differ in fungal community composition as many endophytic and saprophytic fungi are not strictly associated with specific hosts, an aspect discussed by Kowalski and Bilański (2021). Additionally, primers used for ITS region-based metabarcoding may not amplify equally efficiently DNA of all fungi (Cross et al., 2017), whereas fungal isolation on agar may favor fast growing species.

Depending on disease history, common ash forests may differ significantly in *H. fraxineus* infection pressure as was obviously the case for the two stands in our study. Shoot dieback was observed first in 2021 in the subjected healthy stand (unpublished observation), whereas the stand in southern Norway has a disease history that dates to around 2008 (Solheim and Hietala, 2017). Our detection of *H. fraxineus* in petioles collected in 2013 and 2017 from this healthy site would indicate that at that time the infection pressure was probably still below a certain threshold that would be needed for induction of shoot symptoms. This assumption is supported by the low sequence read proportion of *H. fraxineus* in petioles from the healthy stand in comparison to the diseased stand. The length of the local establishment phase of *H. fraxineus* before the onset of shoot symptoms is not widely understood. Based on our data, it appears that this period may last up to 10 years. However, it is worth noting that this forest is located at the northern end of the common ash’s distribution range. This could mean that the pathogen establishment phase is longer here than in ash forests located in the central distribution range with larger ash populations and warmer climate.

Tilman (2004) predicts that successful invaders decrease the abundances of species that are competitively close to them on the trade-off surface. In the present study, the high abundance of *H. fraxineus* and generally low abundance of other species in the order Helotiales, e.g., species of *Tetracladium*, *Crocicreas* and *Chalara*, in petioles from the diseased stand would fit with Tilman’s prediction. The low alpha-diversity indices observed in petioles from the diseased stand in comparison to petioles from the healthy stand at almost all time-points would indicate that *H. fraxineus* suppresses fungal diversity in decomposing ash petioles. The survival of *H. fraxineus* as pseudosclerotia in infected leaf petioles for several years before fruiting body formation (Kirisits, 2015) testifies for a strong ability to defend their saprotrophic niche. The competitive exclusion may initiate already while the leaves are still attached as fungal richness was shown to decline toward autumn in *H. fraxineus* infected ash leaves (Agan et al., 2020).

While the relative read proportions of many fungi differed often strikingly between petioles from the two sites, the prevalent fungal species and the trajectories in their sequence read proportions during overwintering were generally common. The increase in sequence read proportions of fungi in the basidiomycete genus *Mycena* (order Agaricales) toward summer in petioles from both the healthy and diseased stand is noteworthy. For basidiomycetes, some species of *Mycena* have an astonishingly potent carbohydrate active enzyme portfolio on all plant cell wall components (Lange et al., 2021), something that may enable plasticity in feeding on different cell wall carbohydrates according to substrate properties and phase of decomposition.

Chemical and physical traits of litter and functional traits of species within the associated microbial communities are main drivers of litter decomposition (Chomel et al., 2016). The quality of ash leaf litter is obviously affected by *H. fraxineus* as the infected leaves show accumulation of host defense phenolics (Nielsen et al., 2022), which may have potential microbe toxicity or inhibitory effects on microbial enzyme activity. This may partially explain the low level of Agaricomycetes in petioles from the diseased forest as phenolic compounds can suppress oxidative radicals utilized by basidiomycetes to break down the lignocellulose matrix (e.g., Nagy et al., 2022). It is also noteworthy that the correlations between weight loss, FungiQuant Cq values, and total DNA level were low for petioles from the diseased site in comparison to the healthy site. One explanation to this could be that apart from fungi, additional organisms also played an important role in the decomposition of petioles from the diseased site in comparison to petioles from the healthy site. In oak leaves naturally rich in polyphenols, bacterial biomass was shown to increase more vigorously in leaf debris than fungal biomass during the first months following leaf shed (Voříšková and Baldrian, 2013). It remains to be examined whether traits in ash leaf litter derived from host-pathogen interaction prior to leaf shed influence other organisms and ecosystem processes.

The relatively low fungal community diversity in petioles colonized by *H. fraxineus* in the diseased stand and in the reference petioles with *H. albidus* ascomata from the healthy stand would indicate that both fungi have high competence to defend their saprobic niche. This is supported by the fact that both *H. albidus* and *H. fraxineus* form a melanized pseudosclerotial plate outside of the sclerenchyma of leaf vein tissues they inhabit (Baral and Bemmann, 2014; Gross et al., 2014). This layer, completed during the winter months, presumably protects the underlying hyphae against invasion by hyphae of other species (Baral and Bemmann, 2014). Nonetheless, Baral and Bemmann (2014) noted that the pseudosclerotial plates formed by *H. albidus* are usually relatively small, whereas those of *H. fraxineus* typically extend throughout the entire petiole and rachis system. Because of the naturally low population size of *H. albidus* and its small pseudosclerotia size in comparison to those of *H. fraxineus*, this indigenous fungus should not have any negative consequences for overall fungal diversity in ash litter.

Our recent study (Hietala et al., 2022) showed that *H. albidus* can have a similar necrotrophic growth phase in common ash leaves as *H. fraxineus*, but that the transition from endophytic to necrotrophic phase takes place later in the season for *H. albidus* than for *H. fraxineus*. This presumably reflects the differential infection pressure by the two species. The current study showed that these fungi have also similar suppressive effect on fungal diversity in petiole regions they have managed to secure. *Hymenoscyphus albidus* has obviously a similar relation with common ash as *H. fraxineus* has with Asian ash species (Inoue et al., 2019). In Europe, the primary difference between the two fungi would appear to be the high fecundity of *H. fraxineus* in comparison to *H. albidus*; something that could be a result of these species having evolved in contrasting environmental conditions (Hietala et al., 2022). Comparative studies between Asia and Europe are needed to investigate the role of environmental conditions and the microbial community to better understand the great success in life cycle completion by *H. fraxineus* in Europe.

## Data availability statement

The datasets presented in this study can be found in online repositories. The names of the repository/repositories and accession number(s) can be found at: NCBI Bioproject—PRJDB15312.

## Author contributions

AH, CK, HS, IB, LN, M-LH, NN, and VT contributed to methodology, study design, and manuscript writing. AH, CK, LN, and NN contributed to data analysis. All authors contributed to the article and approved the submitted version.

## Funding

This research was supported by the Norwegian Forest Damage Monitoring Program at NIBIO under the project no. 10154, NIBIO internal project Edelframtid, and Independent Research Fund Denmark (DFF|Technology and Production Sciences) under the grant no. 8022-00355B. Open access funding provided by Norwegian Institute of Bioeconomy Research.

## Conflict of interest

The authors declare that the research was conducted in the absence of any commercial or financial relationships that could be construed as a potential conflict of interest.

## Publisher’s note

All claims expressed in this article are solely those of the authors and do not necessarily represent those of their affiliated organizations, or those of the publisher, the editors and the reviewers. Any product that may be evaluated in this article, or claim that may be made by its manufacturer, is not guaranteed or endorsed by the publisher.
