# Supplementary material for: Fungal succession in decomposing ash leaves colonized by the ash dieback pathogen Hymenoscyphus fraxineus or its harmless relative Hymenoscyphus albidus
Source: Front Microbiol. 2023 Apr 14;14:1154344. doi: 10.3389/fmicb.2023.1154344 (PMC10140306; doi:10.3389/fmicb.2023.1154344)
Supplement: Supplementary file 5 [file Data_Sheet_5.docx]

Supplementary Material

Fungal succession in decomposing ash leaves colonized by the ash dieback pathogen *Hymenoscyphus fraxineus* or its native relative *Hymenoscyphus albidus*

Chatchai Kosawang^1,*^, Isabella Børja^2,3^, Maria-Luz Herrero^2^, Nina E. Nagy^2^, Lene R. Nielsen^1^, Halvor Solheim^2^, Volkmar Timmermann^2^ and Ari M. Hietala^4^

*** Correspondence:** Chatchai Kosawang: chko@ign.ku.dk


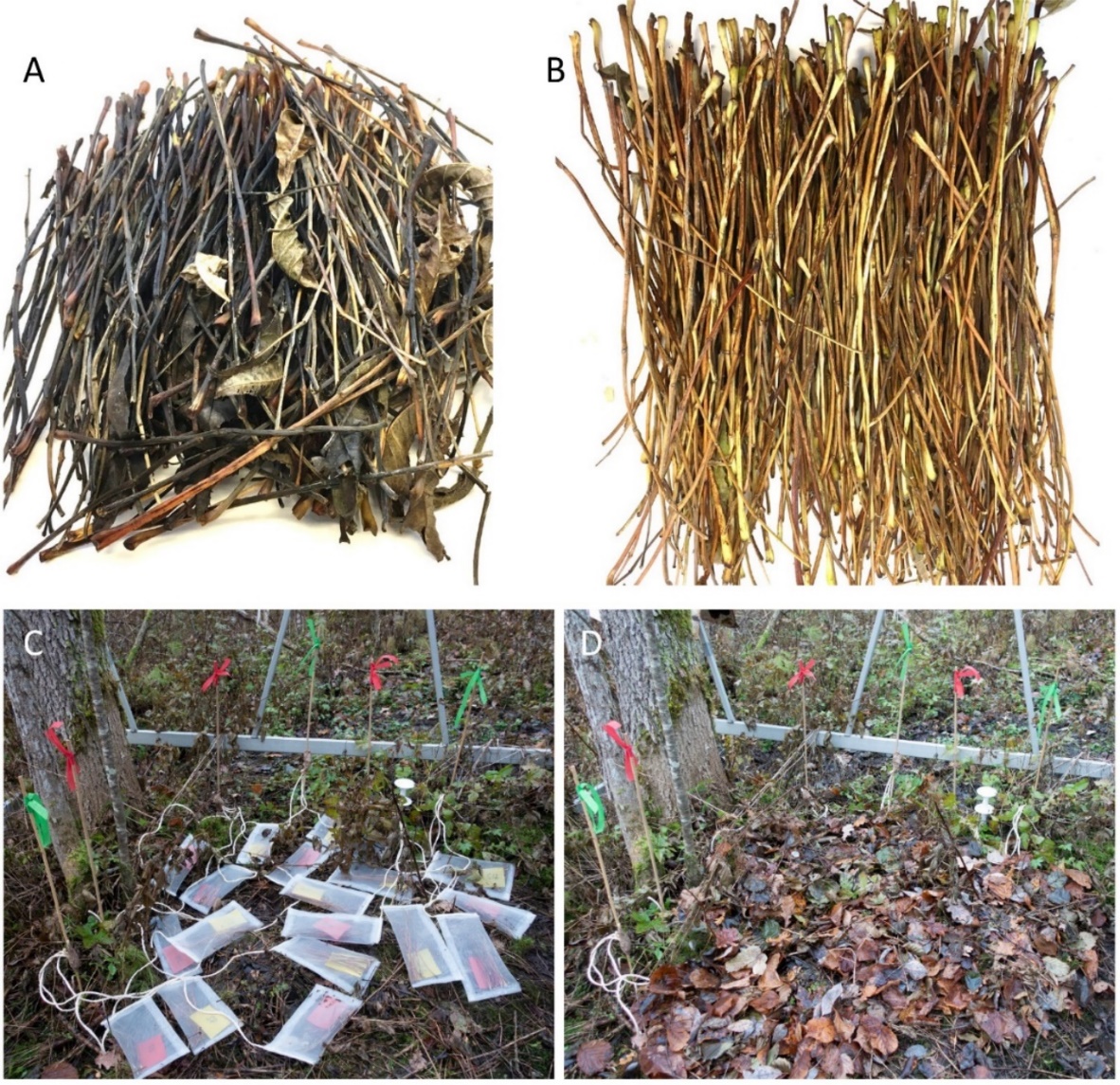


**Supplementary figure 1.** Petioles harvested from (A) Norderås, Ås, in East Norway and (B) Stjørdal in Central Norway in 2017. For the experimental setup, 15 petioles were placed in 12 labelled mesh bags for each site, respectively (red label bags with petioles originating from Norderås and yellow label bags for those from Stjørdal). For control purpose, three bags with petioles from each site were placed in -20°C freezer on 31.10.2017. The remaining bags (altogether 18 bags; 9 bags from with petioles from Norderås and 9 bags from Stjørdal) were spread on the ground (C), anchored with the bamboo sticks and covered with leaves (D). Thereafter, three bags with petioles from each site were collected at three time points: 30^th^ January 2018 ,16^th^ April 2018, and 18^th^ June 2018, and stored at -20°C until further processing.


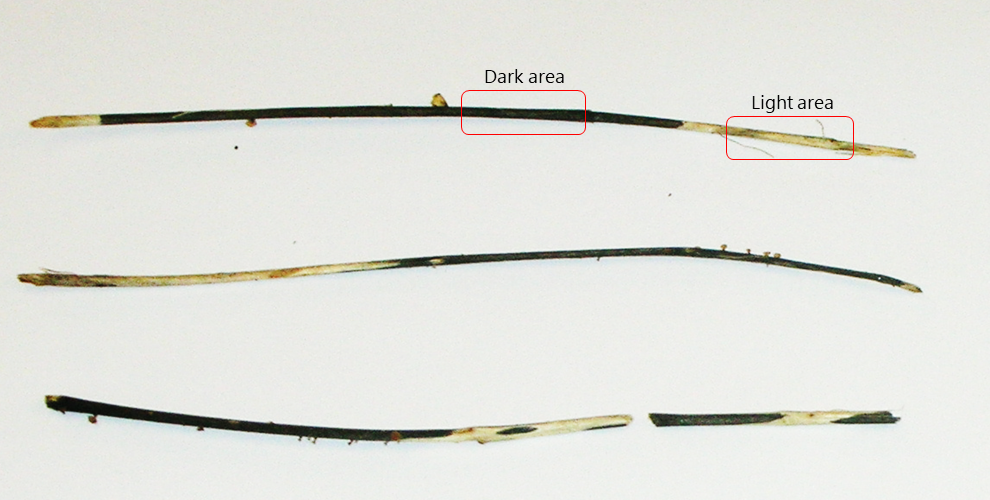


**Supplementary figure 2** Examples of dark and light areas of petioles with *H. albidus* ascomata collected from the healthy site. The dark area contains fully developed pseudosclerotium around the petiole circumference, whereas the light area either showed no pseudosclerotium or only part of the petiole circumference was covered by one pseudosclerotium.


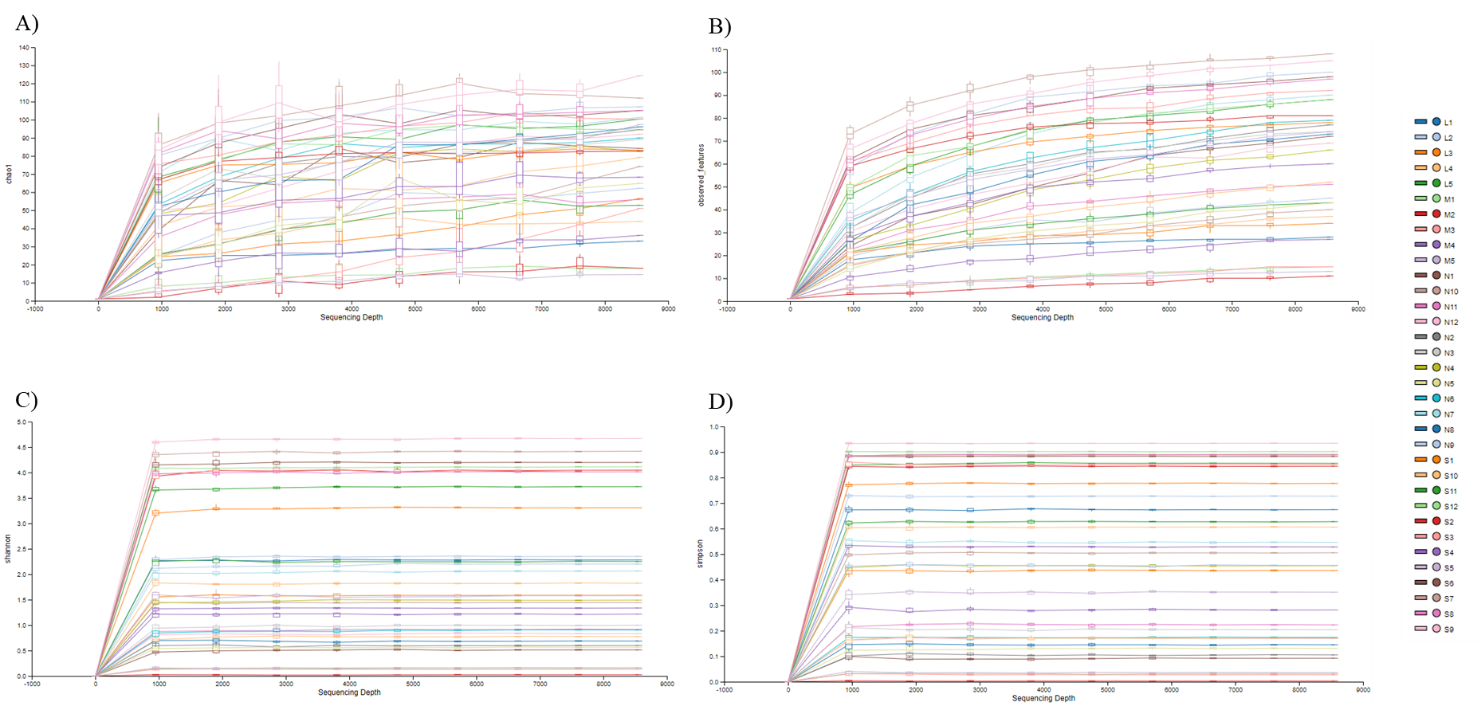


**Supplementary figure 3.** Alpha rarefaction curves of the subsampled sequences (8,557 reads/samples) as measured by (A) Chao1, (B) the observed no. of OTUs, (C) Shannon diversity index and (D) Simpson diversity index. Curves were averaged per time of collection and location.


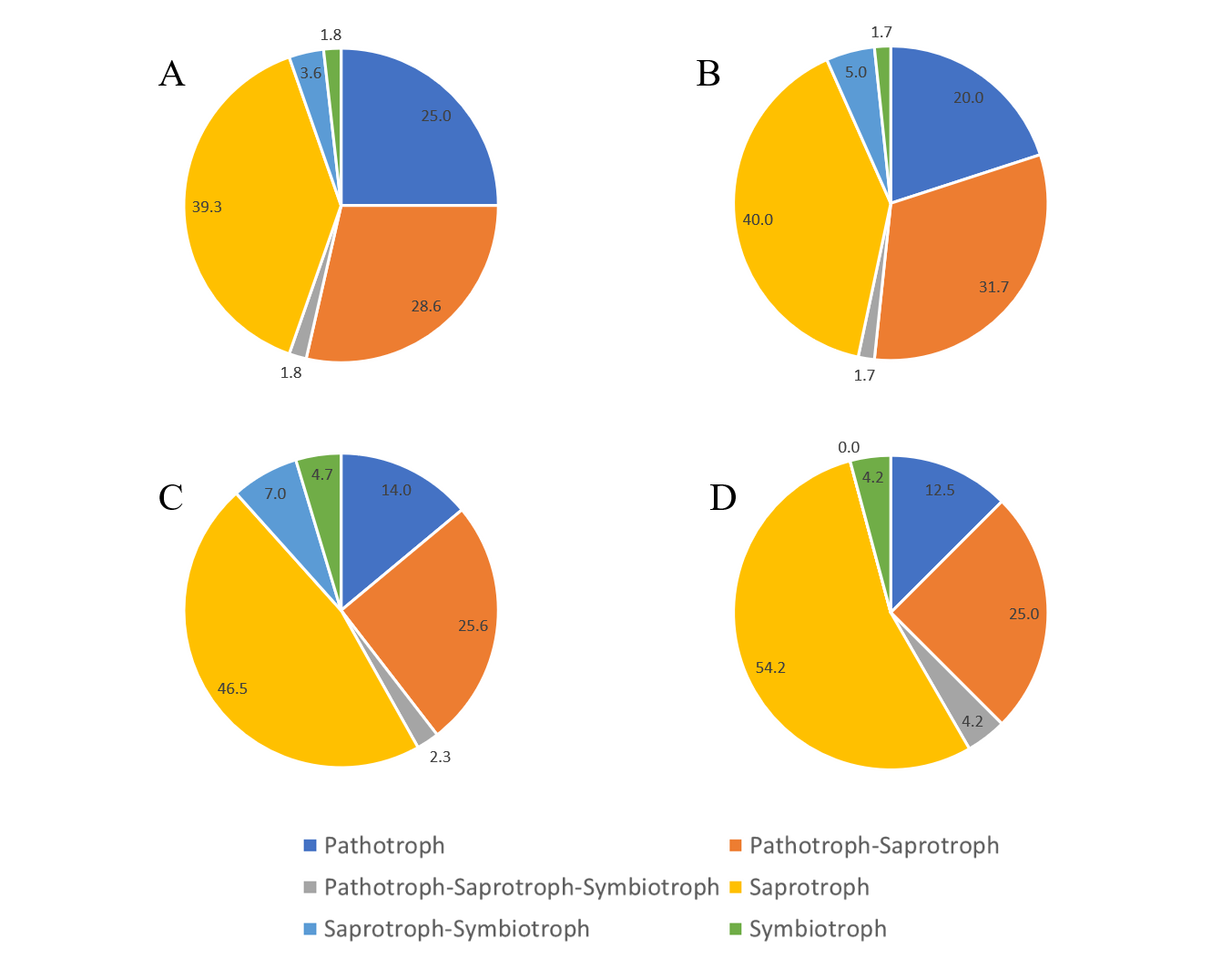


**Supplementary figure 4.** FUNGuild prediction of trophic guilds corresponding to the all-season petiole mycobiomes of (A) Norderås and (B) Stjørdal. The prediction o for the community associated with the light adjacent to pseudosclerotial area and the dark pseudosclerotial area were shown in (C) and (D), respectively. Percentages of such guilds in relation to the total OTUs identified by FUNGuild in each site were shown inside the charts.
